# An Update on Ciguatoxins and CTX-like Toxicity in Fish from Different Trophic Levels of the Selvagens Islands (NE Atlantic, Madeira, Portugal)

**DOI:** 10.3390/toxins13080580

**Published:** 2021-08-20

**Authors:** Pedro Reis Costa, Pablo Estévez, Lucía Soliño, David Castro, Susana Margarida Rodrigues, Viriato Timoteo, José Manuel Leao-Martins, Carolina Santos, Neide Gouveia, Jorge Diogène, Ana Gago-Martínez

**Affiliations:** 1 IPMA—Portuguese Institute of the Sea and Atmosphere, Av. Brasília, 1449-006 Lisbon, Portugal; luciasolino@gmail.com (L.S.); srodrigues@ipma.pt (S.M.R.); 2 CCMAR—Centre of Marine Sciences, Campus of Gambelas, University of Algarve, 8005-139 Faro, Portugal; 3 Biomedical Research Center (CINBIO), Department of Analytical and Food Chemistry, Campus Universitario de Vigo, University of Vigo, 36310 Vigo, Spain; paestevez@uvigo.es (P.E.); dcastro@uvigo.es (D.C.); leao@uvigo.es (J.M.L.-M.); 4 Regional Fisheries Management—Madeira Government, DSI-DRP, Estrada da Pontinha, 9004-562 Funchal, Madeira, Portugal; viriato.timoteo@madeira.gov.pt (V.T.); neide.gouveia@madeira.gov.pt (N.G.); 5 Instituto das Florestas e Conservação da Natureza, IP-RAM, Secretaria Regional do Ambiente, e Recursos Naturais e Alterações Climáticas, Regional Government of Madeira, Rua João de Deus, n.º 12 E/F, R/C-C, 9050-027 Funchal, Madeira, Portugal; carolina.santos@madeira.gov.pt; 6 IRTA, Ctra Poble Nou km 5.5, 43540 Sant Carles de la Ràpita, Spain

**Keywords:** ciguatoxins, Selvagens Islands, seafood safety, *Gambierdiscus*, ciguatera

## Abstract

The Selvagens Islands, which are a marine protected area located at the southernmost point of the Portuguese maritime zone, have been associated with fish harboring ciguatoxins (CTX) and linked to ciguatera fish poisonings. This study reports the results of a field sampling campaign carried out in September 2018 in these remote and rarely surveyed islands. Fifty-six fish specimens from different trophic levels were caught for CTX-like toxicity determination by cell-based assay (CBA) and toxin content analysis by liquid chromatography with tandem mass spectrometry (LC-MS/MS). Notably, high toxicity levels were found in fish with an intermediate position in the food web, such as zebra seabream (*Diplodus cervinus*) and barred hogfish (*Bodianus scrofa*), reaching levels up to 0.75 µg CTX1B equivalent kg^−1^. The LC-MS/MS analysis confirmed that C-CTX1 was the main toxin, but discrepancies between CBA and LC-MS/MS in *D. cervinus* and top predator species, such as the yellowmouth barracuda (*Sphyraena viridis*) and amberjacks (*Seriola* spp.), suggest the presence of fish metabolic products, which need to be further elucidated. This study confirms that fish from coastal food webs of the Selvagens Islands represent a high risk of ciguatera, raising important issues for fisheries and environmental management of the Selvagens Islands.

## 1. Introduction

Ciguatera poisoning (CP) is one of the most common nonbacterial illnesses associated with fish and other seafood consumption worldwide. The illness has been known since the 16th century when ciguateric-like poisonings affected classic sea explorers, such as captains of the Spanish navy in the Gulf of Guinea in 1521 or the crew of captain James Cook in his second voyage to the South Pacific in 1772–1775 [1,2]. CP is widespread in the tropical and subtropical regions and particularly associated with the consumption of reef fish species that have bioaccumulated ciguatoxins (CTXs). These compounds are potent neurotoxins that act at the voltage-gated sodium channels (VGSC) increasing ion permeability and cell disruption leading to persistent neurological impairment [3]. The neurological symptoms include paraesthesia, dysesthesia, vertigo, and sensory abnormalities such as metallic taste, pruritus, arthralgia, myalgia, dental pain, and cold allodynia, a pathognomonic CP symptom that is characterized by burning pain in response to a cold stimulus [4,5,6]. Additionally, a variety of gastrointestinal symptoms including abdominal pain, nausea and vomiting, and cardiovascular symptoms, such as heart rhythm disturbances, may also affect poisoned patients. Some symptoms may become chronic and in extreme cases of severity CP may cause the death of patients [7].

The source of CTXs was linked to benthic dinoflagellates of the genus *Gambierdiscus* in the late 1970s [8,9]. *Gambierdiscus toxicus* was the first species described in the genus and, for a certain period, the only species known of this genus. Although the *Gambierdiscus* genus remains largely understudied, so far, at least 21 species have been described. *Gambierdiscus* and *Fukuyoa* are epibenthic dinoflagellates, which can be ingested by herbivorous fish. Some CTXs are produced by these dinoflagellates, but toxin congeners are products of fish metabolism. *Gambierdiscus* and *Fukuyoa* dinoflagellates are endemic to tropical and subtropical regions which are coincident with the world’s highest incidence areas of ciguatera, namely the South Pacific, the Indian Ocean, and the Caribbean Sea [10]. Nevertheless, increasing occurrence and spread of *Gambierdiscus* to temperate regions has been reported and seems to be favored by the climate warming trends [11,12]. Coincident with CP outbreaks, *Gambierdiscus* have been detected in NE Atlantic subtropical-temperate regions, such as the Canary and Madeira Islands, and even in the Mediterranean Sea [13,14,15,16,17].

The first human poisonings in Europe due to the consumption of autochthonous fish occurred in 2004, in the Canary Islands, Spain [18]. Since then, several outbreaks have been reported in the Canary Islands and also in the nearby Portuguese islands, i.e., Madeira and Selvagens [19]. The first reported outbreak in Portugal dates from 2008 when 11 persons reported CP symptoms after consuming a 30 kg amberjack (*Seriola* sp) caught around the Selvagens Islands [20,21]. The symptoms reported by the crew members matched with symptoms previously reported by nature wardens of the natural park of the Selvagens Islands. CP was retrospectively diagnosed in the six wardens who had consumed fish caught locally [20]. In the following year, CP symptoms were observed in 20–30 people that ingested amberjack purchased in the Canary Islands but caught in the Selvagens Islands [22].

**Figure 1:**
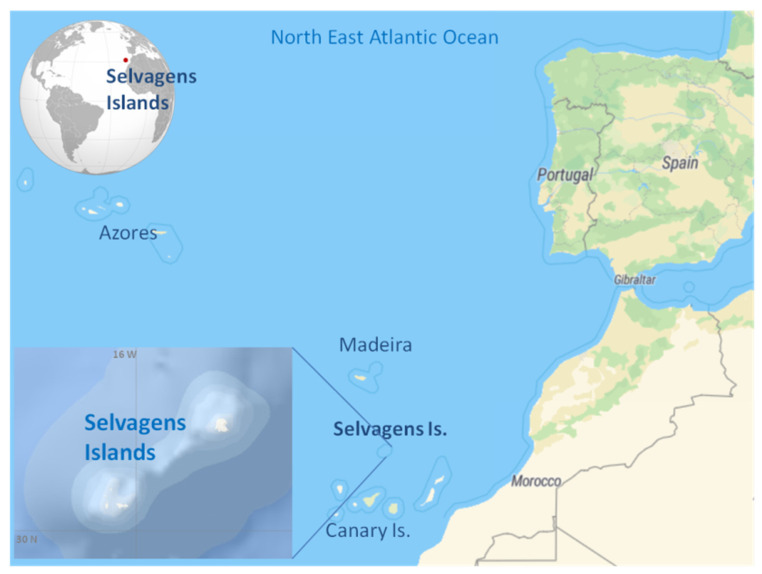
Location of the Selvagens Islands, NE Atlantic, Madeira, Portugal.

The Selvagens Islands are at the southernmost point of the Portuguese maritime area, located in the temperate-subtropical northeast Atlantic, closer to the Spanish Tenerife Island than to the Madeira Island, and 600 km from the African continental coast of Morocco (Figure 1). These small islands have a high fish species diversity and abundance, including important commercial species [23,24]. The total fish biomass has been estimated to be 3.2 times higher in the Selvagens Islands than in the Madeira habitat, and 10 times higher, when only top predators’ biomass were considered [24]. The Selvagens Islands are a unique ecosystem, considered to be one of the last remaining intact ecosystems of the eastern Atlantic Ocean [25].

Despite several human poisonings associated with fish caught in waters surrounding the Selvagens Islands, only a limited number of studies have tried to assess and investigate the presence of CTX in fish from this potential ciguateric hotspot. Following the first human poisoning, in 2008, Otero and colleagues [21] analyzed by liquid chromatography coupled with tandem mass spectrometry (LC-MS/MS) two amberjacks (*S. dumerili* and *S. fasciata*) caught in the Selvagens Islands and described a suite of toxins composed of several CTX, including CTX1B, CTX3C, and C-CTX1. At the same time, Boada et al. [22] reported the presence of C-CTX1 in amberjack specimens associated with human poisonings in Tenerife but that had been caught in the Selvagens Islands. A retrospective analysis carried out by state-of-the-art LC-MS/MS instruments to these specimens confirmed that C-CTX1 was the main toxin analogue but also suggested the presence of several metabolites of this compound [26]. More recently, LC-MS/MS analyses have been performed to assess CTX in fish opportunistically obtained from the Selvagens and Madeira Islands [27]. The results suggested a higher risk of ciguatera for fish caught in the Selvagens Islands and raised the need to better understand the natural contamination of CTX in the coastal food web of this marine protected area. Therefore, a campaign was carried out, in September 2018, on these remote and rarely surveyed islands to collect fish specimens that were representative of several trophic levels to assess CTX-like toxicity by cell-based assay (CBA) and to identify the CTXs by LC-MS/MS.

## 2. Results

### 2.1. Fish Toxicity by CBA

**Figure 2:**
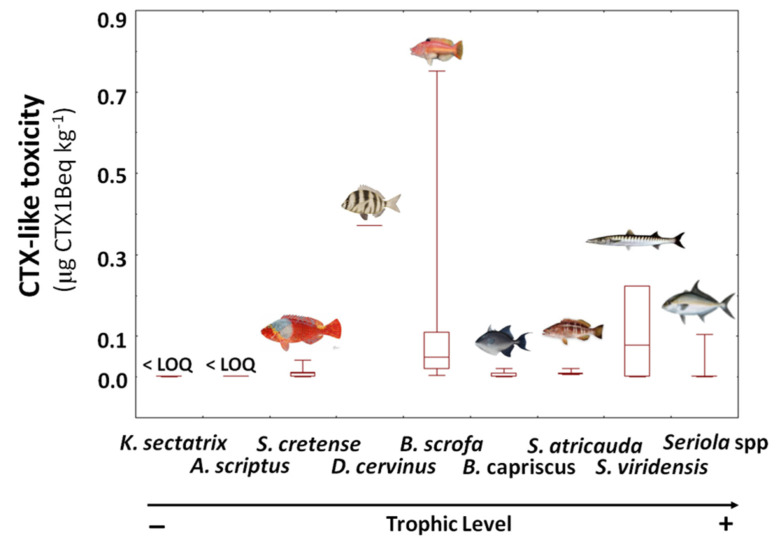
CTX-like toxicity (µg CTX1B equivalent kg^−1^) determined by CBA in fish species collected from the marine food web of the Selvagens Islands during 5–7 September 2018 (median, 25 and 75 quartiles, minimum and maximum, total *n* = 55). See Materials and Methods section for details on samples.

CTX-like toxicity was not detected in specimens at the lowest trophic level, representative of primary consumers of the marine food web, namely Bermuda sea chub *Kyphosus sectatrix* and scribbled leatherjacket filefish *Aluterus scriptus*. However, toxicity was particularly found in fish species with an intermediate position in the food web, such as the parrotfish *Sparisoma cretense*, zebra seabream *Diplodus cervinus,* and barred hogfish *Bodianus scrofa* (Figure 2). From the 14 parrotfish *S. cretense* caught, CTX-like activity was detected in eight specimens ranging from 0.006 to 0.04 µg CTX1B equivalent kg^−1^. The single specimen of zebra seabream *D. cervinus* caught during the field campaign, revealed the second highest level determined, reaching 0.37 µg CTX1B equivalent kg^−1^, highlighting this trophic level of key importance to the CTX dynamics in the Selvagens marine environment. This finding is further supported by the high levels of CTX toxicity found in the 17 barred hogfish *B. scrofa* collected, which varied from 0.04 to 0.75 µg CTX1B equivalent kg^−1^. Although consistently found in each *B. scrofa* analyzed, CTX-like toxicity was not easily correlated with their size or weight (*p* > 0.05).

With a higher position in the food web, grey triggerfish *Balistes capriscus* showed a reduced CTX-like activity in CBA, but toxicity in blacktail comber *Serranus atricauda* was consistently determined in each sample, ranging from 0.006 to 0.02 µg CTX1B equivalent kg^−1^. Highly variable results were observed among the apex predators, the yellowmouth barracuda *Sphyraena viridensis*, and the amberjacks *Seriola* spp, ranging from not detected to considerably high levels (0.22 and 0.10 µg CTX1B equivalent kg^−1^ for *S. viridensis* and *Seriola rivoliana*, respectively).

### 2.2. Toxins Identification and Quantification by LC-MS/MS

With the aim of confirming previous results and characterizing the profile of CTX in fish from the Selvagens Islands, selected samples were analyzed by LC-MS/MS taking into consideration the CTX-like activity measured with the CBA and species trophic level. The presence of Caribbean-CTX1 was identified in the analyzed samples with the highest concentration. The highest level, reaching 0.48 µg kg*^−^*^1^, was determined in a 3.0 kg barred hogfish *B. scrofa* specimen (Table 1). Quantification was performed, following the first detection method described in Section 4.3.3, which was based on the selection of the CTXs [M+Na]^+^ as precursor and product ion. On the one hand, C-CTX1 was consistently detected in all specimens of this species. On the other hand, C-CTX1 or any other CTX analogue was not found in any sample of *Seriola* spp. by LC-MS/MS under the applied conditions. C-CTX1 was also not found in *D. cervinus* and only one of the two barracuda specimens analyzed showed the presence of this toxin and at low levels.

**Figure 3:**
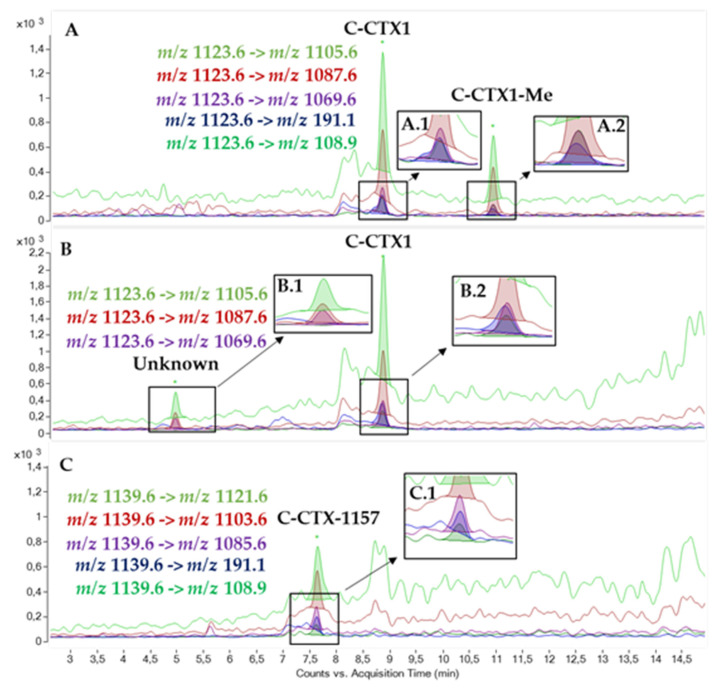
LC-MS/MS chromatogram in MRM mode of: (**A**) Laboratory reference material containing C-CTX1 (8.79 min) and C-CTX1-Me (10.84 min), (A.1) Zoom of C-CTX1 less intense ion transitions: *m/z* 1123.6 [M+H-H_2_O]^+^ -> *m/z* 1069.6 [M+H-3H_2_O]^+^ (purple line), *m/z* 1123.6 [M+H-H_2_O]^+^ -> *m/z* 191.1 (blue line), *m/z* 1123.6 [M+H-H_2_O]^+^ -> *m/z* 108.9 (green line), (A.2) Zoom of C-CTX1-Me less intense ion transitions: *m/z* 1123.6 [M+H-CH_3_-H_2_O]^+^ -> *m/z* 1069.6 [M+H-CH_3_-3H_2_O]^+^ (purple line), *m/z* 1123.6 [M+H-CH_3_-H_2_O]^+^ -> *m/z* 191.1 (blue line), *m/z* 1123.6 [M+H-CH_3_-H_2_O]^+^ -> *m/z* 108.9 (green line); (**B**) C-CTX1 (8.79 min) and an unknown compound (4.94 min) detected in a *Bodianus scrofa*; (**C**) C-CTX-1157 (7.64 min) detected in the *Diplodus cervinus*.

A C-CTX1 methylated congener and additional C-CTX congeners previously detected in a sample of *Seriola fasciata* from the same region and related to a CP case were also monitored by LC-MS/MS [28,29]. The presence of these C-CTX congeners was assessed by monitoring water losses and C-CTX1 specific fragments *m/z* 191.1 and *m/z* 108.9 (Figure 3A). C-CTX1 and a compound at a retention time 4.94 min showing C-CTX1 water losses but not C-CTX1 specific fragments were detected in some *B. scrofa* samples (Figure 3B). Furthermore, a compound matching C-CTX-1157 was detected at a retention time of 7.64 min in the *D. cervinus* in which C-CTX1 was absent (Figure 3C). This compound was detected in one of the fractions obtained after HPLC fractionation, which showed CTX-like activity as indicated in a previous study [29]. Further work is needed to characterize all these congeners, being necessary to obtain the adequate reference materials to be able to accomplish their full characterization.

## 3. Discussion

The Selvagens Islands are a unique ecosystem, listed among the least disturbed islands in the Atlantic Ocean. Despite their small size that implies a small coastal area and their distance to the continental mainland, these islands show high species richness in terms of ichthyofauna, as described by [23]. CTX-like toxicity, as measured by CBA in fish species representative of the several trophic levels, highlighted the presence of toxins in fish at intermediate levels as well as in the top predators. Toxicity was not detected in the two species with the lowest position in the food web, but herbivorous fish, such as the parrotfish *Sparisoma cretense*, and omnivorous species, such as *Diplodus cervinus* and *Bodianus scrofa*, showed notable high toxicity levels. These species commonly show positive relationships in fish assemblage structures and environmental variables, such as seaweed cover and productivity [30]. Further investigation on the presence and abundance of ciguatera-causing dinoflagellates will be presented elsewhere, pointing out the Selvagens Islands as an archipelago with high abundances and biodiversity of marine life playing an important role on the growth and proliferation of *Gambierdiscus* in NE Atlantic (Godinho et al. in prep). The toxicity levels reported in the present study are similar or higher than that observed for groupers and moray eels in the nearby islands of the Canary archipelago [31], raising risks for their consumption.

Although the Selvagens Islands are a marine protected area where fisheries are interdicted, fish inhabiting these islands can migrate and spread CTX to regions in close proximity to the islands. In addition, some fisherman disrespecting fisheries interdictions are occasionally attracted by the abundance and high size fish inhabiting the Selvagens Islands. This was the case of a 4 kg red porgy (*Pagrus pagrus*), a fish species with an intermediate position in the food web, captured by Christmas 2016 that caused CP and was recently reported to contain levels of C-CTX1 as high as 0.76 µg kg^−1^, as determined by LC-MS/MS [26]. In the present study, higher levels of C-CTX1 were found in *Bodianus scrofa* ranging from 0.08 to 0.48 µg kg^−1^. These levels are among the highest determined in Macaronesia and exceeds the U.S. Food and Drugs Administration (FDA) guidance level of 0.1 µg C-CTX1 kg^−1^. Such high levels have been of key importance to fully characterize and confirm fragmentation pathways of C-CTX1 by high resolution mass spectrometry [32].

The present study suggests a higher prevalence and incidence of CTX in fish from the Selvagens Islands than that initially reported by [27]. In this study, two detection methods were used. CBA is a functional assay that provides a toxicological response and LC-MS/MS is a highly selective chemical method that can be used to identify the compounds in the fish matrices. Although a comparison of the advantages and specific issues of these methods is not within the scope of this study, it is important to pay attention to discrepancies regarding CTX toxicity found in the fish top predators, namely the yellow mouth barracuda and the amberjacks*,* and the results obtained by LC-MS/MS below the detection limit. These contrasting results indicate that CTX toxicity of top predators may derive from metabolic fish products of C-CTX1 that have not been elucidated yet. Furthermore, the absence of C-CTX1 and the detection of C-CTX-1157 in a toxic *D. cervinus* showed a possible new toxic profile in which C-CTX1 is not the major contributor to the toxicity. Again, it is important to remember that differences were obtained by these two methods Therefore, further investigations are needed to better understand CTX biotransformation processes, to assess the full suite of toxins in the fish top predators, which are key target species for fisheries in NE Atlantic, and to accurately assess the risk of ciguatera poisoning. This study provides a quite complete evaluation of CTXs in fish (*n* = 56). Nonetheless, for each species or trophic level, the number of fish may be modest and further work increasing number of fish may provide a better estimation of the incidence of CTXs in the different groups.

The Selvagens Islands that are characterized by high abundances and biodiversity of marine life seem to play a role in incubation and proliferation of *Gambierdiscus* (Godinho et al. in prep) and ciguateric fish. Since fisheries, whether commercial or recreational, are forbidden, fish can grow and reach a greater size and can live longer. With a higher lifespan, fish have time to increasingly accumulate and metabolize ciguatoxins. The occurrence of ciguatera in Europe may now pose a new challenge to the management of the marine protected area of the Selvagens Islands.

## 4. Materials and Methods

### 4.1. Fish Sampling

Fifty-six fish specimens belonging to 12 species within different trophic levels were caught between 5 and 7 September 2018 in Selvagem Grande and Selvagem Pequena (Table 2). Estimates of trophic levels were obtained from Fishbase that calculates trophic level from stomach contents data [33]. Fish were caught by means of spearfishing, performed by technicians of the Fisheries Department of Madeira Government, and after the required legal permission of the Madeira Natural Park.

### 4.2. CTX-like Toxicity Measured by Neuroblastoma (Neuro-2a) Cell-Based Assay (CBA)

#### 4.2.1. CTX Extraction for CBA

Fish samples for CBA screening assays were extracted at IPMA, according tothe protocol described elsewhere [34] and with some modifications. A 10 g portion of fish flesh was minced and boiled at 70 °C for 15 min. Once cooled, samples were homogenized with 20 mL of acetone in a Polytron^®^ homogenizer (Fisher Scientific, Porto Salvo, Portugal) at approximately 18,000× *g* for 2 min. Samples were centrifuged for 15 min at 3000× *g* and the pellets extracted again with acetone. Then, the combined supernatants were filtered through a 0.2 μm pore PTFE filter (Whatman, Lisbon, Portugal) with a syringe and evaporated at 60 °C, under vacuum in a rotary evaporator (Fisher Scientific, Porto Salvo, Portugal). The resulting aqueous residue was transferred into a tube and partitioned twice with diethyl ether keeping the ratio 8:2 relative to the amount of residual water. The diethyl ether phases were pooled and evaporated. The dry residue was partitioned with 4 mL of n-hexane and 2 mL of aqueous methanol/H_2_O (4:1, *v*/*v*). The upper phase (hexane) was removed, and the partition repeated twice by adding 4 mL of hexane to the methanolic fraction. The methanolic phase was collected, evaporated, and stored at −20 °C until analysis.

#### 4.2.2. CBA Screening Assay

For cell maintenance, neuro-2a (N2a) cells (ATCC, CCL131) were cultured in 10% fetal bovine serum (FBS) RPMI medium with 1% sodium pyruvate solution (100 mM), 1% l-glutamine solution (200 mM), and 0.5% antibiotic solution (10 mg/mL streptomycin and 1000 U/mL penicillin) (Sigma-Aldrich, St. Louis, MO, USA). Cultures were maintained at 37 °C and 5% CO_2_ in a humid atmosphere incubator (Binder, Tuttlingen, Germany). The day prior to the assay, cells were seeded in a 96-well microplate in 200 μL of 5% FBS-RPMI medium at a density of 35,000 cells per well. Cells were incubated under the same conditions as described for cell maintenance. Every standard (CTX-1B) and sample extract were assayed in triplicate. Half the wells of each microplate were pretreated with ouabain and veratridine corresponding to a final concentration of 1 and 0.1 mM, respectively. Standard solutions and sample extracts were reconstituted in RPMI 5%. Then, samples were serially diluted, and 10 μL added to each well, with (O/V+) and without (O/V-) ouabain and veratridine pretreatment. According to matrix effects estimated without (O/V-), concentrations for the different fish extracts were adjusted to avoid matrix effects. On the next day, cell viability was measured, by means of the MTT test [3-(4,5-dimethylthiadol-2-yl)-2,5-diphenyltetrazolium] (500 μg/mL) [35] and absorbance measured at 570 nm using an automated multi-well scanning. Samples were considered to be positive when cell viability was inhibited in O/V+ wells and unaffected in O/V- wells.

### 4.3. Determination of CTX by Liquid Chromatography with Tandem Mass Spectrometry (LC-MS/MS) Analysis

Sample pretreatment and LC-MS/MS analyses were carried out following the conditions described by [34] and modification performed by [26], and are briefly described below.

#### 4.3.1. Reference Materials

C-CTX1 pure standard (5 ng mL^−1^) was kindly provided by Dr. Robert Dickey (University of Texas, Austin, TX, USA) via Dr. Ronald Manger (Fred Hutchinson Cancer Research Center, Seattle, WA, USA).

The laboratory reference material (C-CTX1 and C-CTX1-Me) extracts from contaminated fish tissue were obtained after HPLC fractionation.

CTX1B (4466 ng mL^−1^) and a qualitative mixture of P-CTXs (CTX1B, 2,3-dihydroxyCTX3C, 51-hydroxyCTX3C, 52-epi-54-deoxyCTX1B/54-deoxyCTX1B, 49-epiCTX3C/CTX3C, and CTX4A/CTX4B) were kindly provided by Prof. Takeshi Yasumoto (Japan Food Research Laboratories, Tokio, Japan).

Calibration was performed by dilution of the stock solution of CTX1B (4466 ng mL^−1^) in methanol. The calibration range selected was from 0.45 to 27.88 ng mL^−1^ (*n* = 5).

C-CTX1 in samples was quantified as CTX1B in the calibration curve and transformed to C-CTX1 eq. with a correction factor previously obtained with C-CTX1 pure standard in the calibration curve.

#### 4.3.2. Sample Pretreatment

First, 15 g of homogenized raw fish muscle was extracted with acetone (2 × 45 mL) using an UltraTurrax^®^. Acetone extracts were combined and evaporated to an aqueous residue which was partitioned with diethyl ether (2 × 15 mL). Diethyl ether layers were combined and evaporated to dryness. The solid residue was dissolved in 90% methanol (4.5 mL) and defatted with hexane (9 mL). The resulting methanolic fraction was dried and resuspended in ethyl acetate (2 mL) for clean-up procedures. A Florisil cartridge (J. T. Baker, 500 mg, Center Valley, PA, USA) was conditioned with ethyl acetate (3 mL) and the sample loaded into the cartridge. The cartridge was washed with ethyl acetate (3 mL) and C-CTXs eluted with ethyl acetate/methanol (9:1, *v:v*) (5 mL) and ethyl acetate/methanol (3:1, *v:v*) (5 mL). The second fraction, containing most of the toxins was further dried under nitrogen at 50 °C, and then dissolved in 60% methanol (2 mL) for a second clean-up step. Sample was loaded into a C18 cartridge (SUPELCLEAN, Supelco, 500 mg, Bellefonte, PA, USA) previously conditioned with 60% methanol (3 mL). The cartridge was washed with 60% methanol (3 mL) and CTXs were eluted with 90% methanol (5 mL). The final eluate was dried under nitrogen at 50 °C and dissolved in 0.5 mL of MeOH LC-MS grade filtering (Syringe Driver filter Unit, Millex^®^-CV 0.22 μm, 13 mm, Millipore, Billerica, MA, USA) prior to LC-MS/MS analysis.

#### 4.3.3. LC-MS/MS Analysis

An Agilent 1290 Infinity LC system coupled to an Agilent 6495 iFunnel Triple Quadrupole LC-MS (Agilent Technologies, Waldbronn, Germany) were used for the LC-MS/MS analyses. Two different methods of analysis were used in order to obtain the confident identification, quantification, and confirmation of the toxins responsible for the contamination:

The first method was used for the sensitive identification and quantification of the CTXs. The chromatographic separation was performed in a Poroshell 120 EC-C18 (3.0 × 50 mm, 2.7 µm, Agilent, Santa Clara, CA, USA) column set at 40 °C. The mobile phases were: (A) 0.1% formic acid and 5 mM ammonium formate in water (J. T. Baker, Center Valley, PA, USA) and (B) methanol (Merck KGaA, Darmstadt, Germany). The gradient of mobile phase started at 78% of B increasing to 88% of B in 10 min, and holding for 5 min to finally increase to the 100% of B at 15.01 min washing the column for 3 min before reducing to the initial conditions at 18 min equilibration for 4 min before the following injection. The injection volume was set at 1 µL and the flow rate 0.4 mL/min.

The MS instrument operated in ESI^+^ in multiple reaction monitoring (MRM) mode monitoring as precursor and product ion at 40 eV the sodium adduct [M+Na]^+^ of the different CTXs with reference material available: CTX1B (*m/z* 1133.6 [M+Na]^+^ -> *m/z* 1133.6 [M+Na]^+^), C-CTX1 (*m/z* 1163.7 [M+Na]^+^ -> *m/z* 1163.7 [M+Na]^+^), C-CTX1-Me (*m/z* 1177.6 [M+Na]^+^ -> *m/z* 1177.6 [M+Na]^+^), 2,3-dihydroxyCTX3C (*m/z* 1079.6 [M+Na]^+^ -> *m/z* 1079.6 [M+Na]^+^), 51-hydroxyCTX3C (*m/z* 1061.6 [M+Na]^+^ -> *m/z* 1061.6 [M+Na]^+^), 52-epi-54-deoxyCTX1B/54-deoxyCTX1B (*m/z* 1117.6 [M+Na]^+^ -> *m/z* 1117.6 [M+Na]^+^), 49-epiCTX3C/CTX3C (*m/z* 1045.6 [M+Na]^+^ -> *m/z* 1045.6 [M+Na]^+^), and CTX4A/CTX4B (*m/z* 1083.6 [M+Na]^+^ -> *m/z* 1083.6 [M+Na]^+^).

Source and interface settings were: Drying gas 15 L/min of N_2_ at 290 °C; sheath gas 12 L/min of N_2_ at 400 °C; nebulizer gas, N_2_ at 50 psi; capillary voltage, 5000 V; nozzle voltage: 300 V; fragmentor potential 380 V.

The second method was used to confirm the presence of C-CTX1 and specific C-CTX congeners. The chromatographic separation was performed in a Poroshell 120 EC-C18 (2.1 × 100 mm, 2.7 μm, Agilent USA) column set at 40 °C. The mobile phases were: (A) 0.1% formic acid and 5 mM ammonium formate in water (J. T. Baker, Center Valley, PA, USA); (B) acetonitrile (Merck KGaA, Darmstadt, Germany). The gradient of mobile phase started at 35% of B for 1 min increasing to 80% of B in 15 min, increasing in 1 min to a 95% of B and holding for 5 min to finally return to the initial conditions at 24 min equilibrating for 4 min before the following injection. The injection volume was set at 5 µL and the flow rate 0.4 mL/min.

The MS instrument operated in ESI^+^ in MRM mode monitoring C-CTX1 water losses ([M+H-nH_2_O]^+^) and specific fragments *m/z* 191.1 and *m/z* 108.9. The MS/MS ion transitions for the C-CTXs detected are summarized in Table 3. Source and interface settings were: Drying gas 16 L/min of N_2_ at 250 °C; sheath gas 12 L/min of N_2_ at 400 °C; nebulizer gas, N_2_ at 15 psi; capillary voltage, 5000 V; nozzle voltage 1000 V; fragmentor potential 380 V.

## Figures and Tables

**Table 1:** Concentration of Caribbean Ciguatoxin-1 (C-CTX1) determined by LC-MS/MS in selected fish species caught in the Selvagens Islands (NE Atlantic, Portugal) in September 2018.

Species	*n*	C-CTX1 (µg kg^−1^)
*Bodianus scrofa*	10	0.08–0.48
*Balistes capriscus*	2	<LOQ–0.09
*Diplodus cervinus*	1	<LOQ
*Seriola* spp.	5	<LOQ
*Sphyraena viridensis*	2	<LOQ–0.14

LOQ, limit of quantification (0.015 µg kg^−1^).

**Table 2:** Length and weight of fish specimens caught in the coastal food web of the Selvagens Islands habitat.

Trophic Level *	Species	Common Name	*n*	Length Range (mm)	Weight Range (g)
2.0	*Kyphosus sectatrix*	Bermuda sea chub	5	420–530	1178–2387
2.8	*Aluterus scriptus*	Scribbled leatherjacket filefish	1	640	2417
2.9	*Sparisoma cretense*	Parrotfish	14	280–350	409–849
3.0	*Diplodus cervinus*	Zebra seabream	1	520	2840
3.5	*Bodianus scrofa*	Barred hogfish	16	390–530	1122–3010
4.1	*Balistes capriscus*	Grey triggerfish	4	350–490	585–2640
4.3	*Serranus atricauda*	Blacktail comber	5	250–300	193–336
4.3	*Sphyraena viridensis*	Yellowmouth barracuda	3	850–1170	2246–5955
4.5	*Seriola dumerili*	Greater amberjack	1	850	5656
4.5	*Seriola fasciata*	Lesser amberjack	2	490–620	1602–2795
4.5	*Seriola rivoliana*	Longfin yellowtail	2	600–1060	12,308–2536
4.5	*Seriola* spp	Amberjack	1	460	1163

* Estimated trophic level (TL) as expressed in Fishbase (2021). TL = 2.0 is at the base of the consumers food web, with their diet composed mainly of detritus and plants, and TL = 5.0 is a top predator fish.

**Table 3:** MS/MS conditions for the confirmation of specific C-CTXs.

Compound	Precursor Ion (Q1)	Product Ion (Q3)	CE (eV)
C-CTX-1157	[M+H-H_2_O]^+^ *m/z* 1139.6	[M+H-2H_2_O]^+^ *m/z* 1121.6	15
	[M+H-H_2_O]^+^ *m/z* 1139.6	[M+H-3H_2_O]^+^ *m/z* 1103.6	30
	[M+H-H_2_O]^+^ *m/z* 1139.6	[M+H-4H_2_O]^+^ *m/z* 1085.6	30
	[M+H-H_2_O]^+^ *m/z* 1139.6	*m/z* 191.1	41
	[M+H-H_2_O]^+^ *m/z* 1139.6	*m/z* 108.9	52
C-CTX1 and isomers	[M+H-H_2_O]^+^ *m/z* 1123.6	[M+H-2H_2_O]^+^ *m/z* 1105.6	25
	[M+H-H_2_O]^+^ *m/z* 1123.6	[M+H-3H_2_O]^+^ *m/z* 1087.6	29
	[M+H-H_2_O]^+^ *m/z* 1123.6	[M+H-4H_2_O]^+^ *m/z* 1069.6	37
	[M+H-H_2_O]^+^ *m/z* 1123.6	*m/z* 191.1	41
	[M+H-H_2_O]^+^ *m/z* 1123.6	*m/z* 108.9	52
	[M+H-CH_3_-H_2_O]^+^ *m/z* 1123.6	[M+H-CH_3_-2H_2_O]^+^ *m/z* 1105.6	25
	[M+H-CH_3_-H_2_O]^+^ *m/z* 1123.6	[M+H-CH_3_-3H_2_O]^+^ *m/z* 1087.6	29
C-CTX1-Me	[M+H-CH_3_-H_2_O]^+^ *m/z* 1123.6	[M+H-CH_3_-4H_2_O]^+^ *m/z* 1069.6	37
	[M+H-CH_3_-H_2_O]^+^ *m/z* 1123.6	*m/z* 191.1	41
	[M+H-CH_3_-H_2_O]^+^ *m/z* 1123.6	*m/z* 108.9	52
